# Impact of antenatal exposure to a mixture of endocrine disruptors on attentional and executive functions in children

**DOI:** 10.1210/jendso/bvag057

**Published:** 2026-03-17

**Authors:** Christophe Barrea, Patrice Dufour, Catherine Pirard, Corinne Charlier, Fanny Brévers, Anne-Simone Parent, Laurence Rousselle

**Affiliations:** Department of Paediatrics, University of Liege (ULiege), CHU, Liege 4000, Belgium; GIGA Neurosciences, Neuroendocrinology Unit, University of Liege (ULiege), CHU, Liege 4000, Belgium; Laboratory of Clinical, Forensic and Environmental Toxicology, University of Liege (ULiege), CHU, Liege 4000, Belgium; Laboratory of Clinical, Forensic and Environmental Toxicology, University of Liege (ULiege), CHU, Liege 4000, Belgium; Laboratory of Clinical, Forensic and Environmental Toxicology, University of Liege (ULiege), CHU, Liege 4000, Belgium; Research Unit for a Life-Course Perspective on Health and Education, University of Liege (ULiege), Liege 4000, Belgium; Department of Paediatrics, University of Liege (ULiege), CHU, Liege 4000, Belgium; GIGA Neurosciences, Neuroendocrinology Unit, University of Liege (ULiege), CHU, Liege 4000, Belgium; Research Unit for a Life-Course Perspective on Health and Education, University of Liege (ULiege), Liege 4000, Belgium

**Keywords:** endocrine-disrupting chemicals, persistent organic pollutants, mixture, cognition, preschool, sex-stratified effect

## Abstract

**Context:**

Numerous studies indicate negative associations between early life exposure to endocrine-disrupting chemicals and various aspects of neurodevelopment. However, few have focused on specific cognitive processes. Additionally, toxicants are often analyzed individually, without accounting for their combined effects.

**Objective:**

This study aimed at investigating the impact of prenatal exposure to a mixture of endocrine disruptors on attention and executive functions in young children and comparing their effects with those reported in the literature.

**Methods:**

Two polychlorinated biphenyls (PCBs) and 4 perfluoroalkyl substances (PFASs) were measured in the cord blood from 55 children enrolled in a longitudinal Belgian cohort study. At 6 years of age, attentional and executive functions were assessed using specific neuropsychological tests. Associations between a mixture of toxicants and cognitive performance were analyzed using the principal components approach and weighted quantile sum regression, while accounting for sex differences.

**Results:**

Higher prenatal exposure to PCB mixtures was significantly associated with an increased number of omissions in the Divided Attention test. In sex-stratified analyses, this association remained significant but was observed only in boys. Additionally, boys exhibited reduced working memory and planning abilities following exposure to a mixture of PCBs and PFASs. In contrast, antenatal exposure to a mixture of PCBs and PFASs in girls was associated with reduced behavioral regulation, including inhibition control, as assessed by parent-reported questionnaires screening executive functioning in daily life.

**Conclusion:**

These results support associations between antenatal exposure to a mixture of endocrine disruptors and attention and executive development, emphasizing a sex-specific effect.

Attention is a cognitive process that allows us to choose and concentrate on relevant stimuli. It allows people to focus on information to create memories and to avoid distractions, so that they can focus on and complete specific tasks [1, 2]. Attention is closely related to executive functions (EFs), which encompass higher-order neurocognitive processes, including inhibition, working memory (WM), and cognitive flexibility [3]. Executive functions are recruited for controlled action in new and/or complex situations, especially when well-learned, routine action schemas are no longer adequate to meet the demands of the task [4].

Both attentional and EFs play a critical role in almost every area of life, including school, work, and relationships. They are essential skills for mental and physical health, academic, and personal success, as well as for cognitive, social, and psychological development [5]. They are also key components of behavioral and emotional regulation [6].

Deficits in attentional and executive functioning are found in many conditions, the most common being the attention-deficit/hyperactivity disorder (ADHD). This condition is characterized by a persistent pattern (at least 6 months) of inattention and/or hyperactivity-impulsivity that has a direct negative impact on academic, occupational, or social functioning [7]. Inattention refers to significant difficulty in sustaining attention to tasks that do not provide a high level of stimulation or frequent rewards, distractibility, and problems with organization. Hyperactivity refers to excessive motor activity and difficulties with remaining still, most evident in structured situations that require behavioral self-control. Impulsivity is a tendency to act in response to immediate stimuli, without deliberation or consideration of the risks and consequences. The disorder typically starts in childhood and can persist into adolescence and sometimes adulthood [8].

The prevalence of ADHD in children and adolescents is estimated to be 7.2% worldwide and seems to have increased in recent years [9, 10]. Improved diagnostic criteria and awareness of the disease might be responsible for the increased detection of ADHD cases, but they do not alone explain the rise observed.

The underlying causes of ADHD are most likely interactions between genetic and nongenetic factors [11, 12]. While the role of heritability in the etiology of ADHD is well documented, knowledge about how environmental factors may affect the development of ADHD is still scarce. For example, exposure to environmental toxics, such as lead, was found to be associated to the risk of ADHD [13, 14] and executive dysfunction [15]. Since genes are stable over years, increased exposure to environmental pollutants, such as endocrine disruptors (EDCs), might contribute to the rising prevalence of ADHD [16].

According to the World Health Organization, “EDCs are exogenous substances present in the environment which alter the functions of the endocrine system, causing adverse health effects in an intact organism, its offspring, or a (sub) population” [16]. These chemicals include polychlorinated biphenyls (PCBs) and perfluoroalkyl substances (PFASs), which are/were extensively used in industry and are present in the serum of the general population in Europe and in the United States [17]. Some of them are of particular concern due to their bioaccumulating properties, which lead to high concentrations in humans. These toxicants cross the placenta and accumulate in the fetus [18]. Given that the brain development is particularly susceptible to disruption by environmental pollutants during fetal period, it is of importance to investigate the association between exposure to these pollutants during this sensitive period and ADHD [19].

Animal studies of prenatal and postnatal exposures to EDCs have demonstrated a relationship between antenatal toxicant exposure and ADHD-like symptoms, including motor hyperactivity and inability to inhibit responses [20-22]. A variety of studies also used animal models to assess the effects of EDCs exposure on EFs [23]. In humans, numerous studies also provided evidence of a negative correlation between various components of neurodevelopment, including intelligence and motor functions, and antenatal exposure of EDCs [24, 25]. However, few studies focused on specific cognitive processes. This is particularly true as most investigators rely on broad measures of global cognitive functioning, which consist of composite tests assessing multiple cognitive processes. While both epidemiological and experimental evidence suggested that PCBs and PFASs were developmental neurotoxicants [26], research exploring their effects on attentional and executive skills in children remained limited and inconsistent [27-30]. Various methodologies have been used, including neuropsychological tests, clinical evaluations, and behavioral questionnaires, which provide indirect subjective measures of cognitive outcomes (Tables SA and SB [31]). Moreover, most studies examining associations between prenatal EDCs exposure and cognitive neurodevelopment in children relied on single-chemical models, while mixture analyses have been increasingly encouraged to assess additive or synergistic effects, providing a more realistic representation of the toxicity associated with chemical mixtures in natural environments [32-35]. Finally, some studies demonstrated sex-specific associations between prenatal exposure to EDCs and neurodevelopmental outcomes in children [33, 36].

Cognitive impairment resulting from early-life exposure to endocrine-disrupting chemicals (EDCs), even at moderate levels, can have far-reaching socio-economic consequences for the general population [37]. Among these, deficits in attentional functions represent a substantial component of the neurodevelopmental impact, with an estimated cost in Europe ranging from €1.2 to 2.9 billion annually [38]. Despite regulatory efforts that have led to a reduction in the levels of certain toxicants, their persistent presence in the environment, bioaccumulation in human tissues, and occurrence in materials continue to pose public health concerns. Therefore, the present study aimed at investigating the associations between prenatal exposure to PCBs and PFASs and attentional and EFs in children at 6 years of age. Using 2 statistical approaches adapted for mixture analyses, we examined cognitive outcomes in boys and girls, both together and separately.

## Methods

### Study participants

Participants were part of the “Effet des Polluants Organiques Persistants sur l’Evolution des Enfants” cohort described elsewhere [24].

Briefly, every woman admitted for delivery at the University Hospital of Liege (Belgium) between 2014 and 2016 was asked to participate in a study. Umbilical cord blood samples of their newborn were collected, centrifugated, and stored at −80 °C immediately after delivery. Inclusion criteria include the absence of prematurity and congenital disease.

Of the 212 original participants, 77 gave their consent for cognitive evaluation of their child at 6 years of age (M = 5.75; standard deviation [SD] = 0.34), before starting elementary school. Finally, 55 of them had enough cord blood to perform EDC measurement and were selected for the present study.

### Exposure

Among the 27 EDCs initially measured in cord blood samples, our study focused on the 6 chemicals detected in at least 50% of the population [39]: 2 PCBs (PCB-153 and PCB-180) and 4 PFASs (perfluorooctane sulfonate [PFOS], perfluorooctanoic acid [PFOA], perfluorohexane sulfonate [PFHxS], and perfluorononanoic acid [PFNA]). The detailed analytical method has been described by Dufour et al [40].

For each EDC, the limit of quantification (LOQ) was established during the validation process and defined as the lowest concentration measurable with a maximum uncertainty not exceeding 40%. Following the recommendations of Lubin et al [41], values below the detection limit were replaced using multiple imputation techniques to generate 5 imputed datasets (K = 5) [42, 43]. To reduce variability and satisfy the normality assumptions required by our statistical models, a logarithmic transformation was applied to the data.

### Cognitive abilities assessment

The attentional functions were examined using a set of 3 computerized tasks designed to assess the attentional components varying along the dimension of selectivity, specifically targeting selective attention in both Auditory Sustained Attention (ASA) and Visual Sustained Attention (VSA) modalities and Divided Attention (DA), in accordance with the Van Zomeren and Brouwer model [1]. These tasks were inspired from the Test of Attentional Performance [44] and implemented in a game called the “Magical Piano” designed and presented using the PsychoPy3 program (Open Science Tools, 2019).

**Figure 1:**
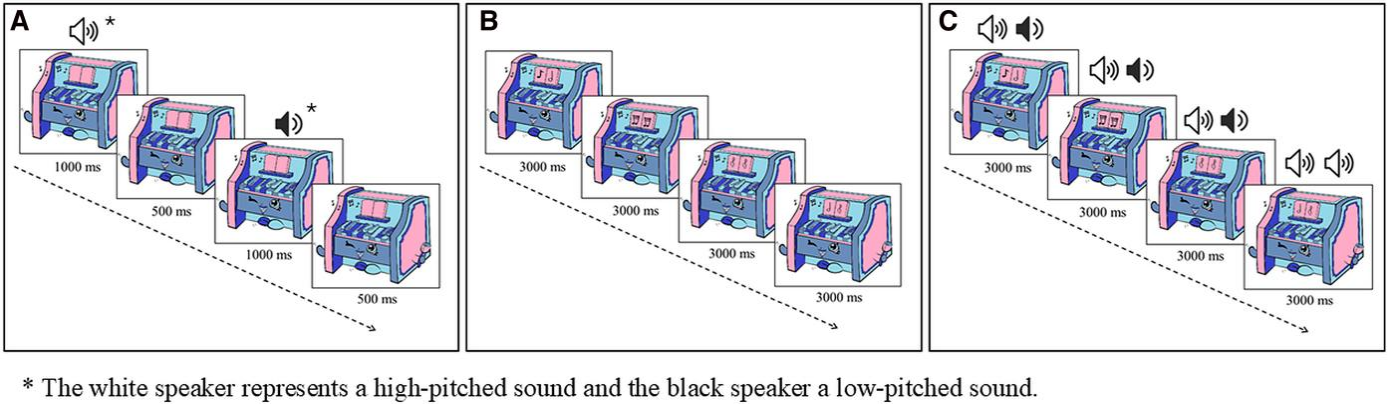
Schematic illustration of the 3 attentional tests of the Magical Piano game: selective auditory attention task (A), selective visual attention task (B), and divided attention task (C); the white speaker represents a high-pitched sound and the black speaker a low-pitched sound.

Selective attention tasks required children to select target information from a set of distractors either in the auditory or visual modality [1]. In the auditory selective attention test (Fig. 1A), the child had to identify the succession of 2 identical tones by pressing a button as quickly as possible. The sounds followed one another at 1.5-s intervals (1000 ms of stimulus presentation and 500 ms interval between 2 stimuli). Seventy tone pairs were played, and 12 (17%) required a response. The total test duration was 3 minutes and 30 s. In the visual selective attention test (Fig. 1B), the child was asked to react as quickly as possible by pressing a button when the 2 notes depicted on the score were identical. The images changed every 3 s. 70 visual stimuli were presented, and 12 of them (17%) required a response. The total test duration was 3 minutes and 30 s.

Finally, DA was measured using a DA test requiring attention to be allocated to different modalities at the same time (Fig. 1C). The child had to perform both the visual and auditory selective attention tasks simultaneously, reacting to visual stimuli (when the 2 notes were identical) and auditory stimuli (when 2 tones of the same pitch followed one another). As in the selective attention tests, the visual stimuli changed every 3 s, while the auditory stimuli occurred at 1.5 s intervals. Therefore, 2 tones were played during the presentation of a single visual stimulus. The test included a total of 70 visual stimuli, of which 8 (12%) required a reaction, and 70 pairs of tones, of which 8 (12%) required a response. The total duration of the test was 3 minutes and 30 s.

Executive functions were examined using both questionnaires and cognitive tests. First, Behavior Rating Inventory of Executive Function (BRIEF) [45] was administered to provide a broad assessment of executive functioning in daily life. This valid and reliable parent-reported questionnaire was based on 8 core subscales used to calculate 2 indexes: the Behavioral Regulation Index (sum of the inhibition, flexibility, and emotional control subtests) and the Metacognition Index (sum of the initiation, working memory, planification, organization and auto-regulation subtests). The combination of these 2 indexes constituted the global score (mean = 50, SD = 10), higher scores indicated lower level of executive functioning.

The EFs were also examined using a set of 4 cognitive tasks assessing verbal and motor inhibition, flexibility and planification. Response time (RT) and accuracy (number of correct and incorrect responses) were recorded for each task, with RT analyses restricted to correct trials and outliers (> ±2 SD) excluded. Verbal inhibition and flexibility abilities were evaluated using the Stroop Fruits task [46]. In the naming condition, the child had to name the color of successive rectangles (red, green, and yellow) as quickly as possible. This condition measured color naming speed, and the scores obtained were used as covariates in the analyses of Stroop Inhibition and Flexibility to control for interindividual naming speed differences. In the second condition, the child had to quickly name the real color of the fruits presented in black and white to ensure the child knew the colors of the fruits. In the third condition assessing verbal inhibition, each presented fruit was drawn in a color different from its natural one (green banana, red pear, and yellow strawberry). The child's task was to name the real color of the 45 fruits (7 per line) as quickly as possible. This test was supplemented by a last condition assessing reactive flexibility. It was identical to the previous condition except that the child was asked to switch between tasks, alternating between inhibition and non-inhibition trials, depending on whether the fruits were framed or not. For framed fruits (10 fruits on the whole test, 2 per line), the child had to name the natural color of the fruit, as in the inhibition condition (ie, saying “yellow” for a green banana), while for the unframed trials, the child was asked to name the color in which the fruit is drawn (ie, saying “green” for a green banana). Each correct response was credited with 1 point, for a total score of 45 in verbal inhibition and of 45 for the flexibility subtest.

The Go/NoGo task was used to assess motor inhibition skills. This computerized task adapted from the Test of Attentional Performance [47] was implemented using E-Prime 2.0 (Psychology Software Tools, 2012). Two types of stimuli appeared on a black screen: a cross (×) or a plus sign (+). The child had to react only to the appearance of the cross by pressing a button as quickly as possible. No response was required when the plus sign appears, meaning the motor response had to be inhibited. The stimuli appeared for 200 ms, and the interval between stimuli was 3 s. A total of 45 stimuli were presented during the task, including 30 trials requiring a reaction (2/3 of items “Go” to increase demands in inhibitory control). The total test duration was ∼1 minute and 20 s. Each correct response was credited with 1 point, for a total score of 45 in motor inhibition. Finally, the Spatial Planning subtest from the Kaufman Assessment Battery for Children Second Edition (K-ABC-2) assessment battery was administered to evaluate planning skills. The child was instructed to help a dog reaching its bone on a matrix board while avoiding obstacles, making as few moves as possible. Each correct response was credited with 2 or 1 points, for a total score of 38 in planning subtest.

### Covariates

As ADHD results from complex interactions between genetic, environmental, and social factors [48], numerous covariates were considered based on Directed Acyclic Graph theory [49], including parental education and smoking status, maternal age, and parity (Fig. SC [31]). The age of the child was also included, as it has a significant influence on the outcomes. Certain parameters, such as peri/antenatal diseases and the presence of chronic conditions, were excluded since they were part of the study's exclusion criteria. Additionally, other covariates, such as alcohol consumption during pregnancy and breastfeeding, were selected a priori based on evidence from previous literature supporting their substantial influence on cognitive development [50, 51].

Information on potential confounders was obtained from medical records and a questionnaire completed during the first study visit.

To identify significant variables in the association between exposures and outcomes (*P* < .20) [52], Student's *t*-tests were used for binary variables, and univariate regression models were applied to evaluate the effects of continuous variables on outcome measures (Table SD [31]). Multiparity, parental smoking, educational status, and the age of the child during testing influenced at least one parameter across all attentional tests. Maternal age was associated with the number of errors in the VSA test and ASA test, respectively. The sex of the child and his birth weight impacted the error rates of omissions (Om) in DA test. For EFs, parity, maternal smoking status, and the child's naming speed influenced performance on the Stroop Inhibition test. Parental smoking status was associated with the number of errors in the Go/NoGo test, while maternal parity and age were linked to the Spatial Planning score. Breastfeeding duration was associated with the global and index scores of the BRIEF questionnaire. Parity was also associated with the global and behavioral regulation scores, while the father's smoking status and the age of mother influenced the global and metacognition scores, respectively. Finally, no significant correlations were observed between the Stroop Flexibility test and any of the covariates studied.

### Statistical analysis

Given the relatively small sample size and the risk of collinearity due to strong correlations between certain toxicants, 2 statistical models were employed to investigate the associations between attentional and executive scores and prenatal exposure to a mixture of EDCs. Firstly, principal component analysis (PCA) was performed on the matrix of log-transformed biomarkers to identify a reduced number of uncorrelated components (Comp) representing exposure to substances [53]. Components explaining at least 50% of the cumulative variance were selected [54] and used to predict outcomes of interest through multiple linear regression models [55]. Secondly, Weighted Quantile Sum (WQS) approach was applied to evaluate associations between correlated joint exposures and the health outcomes of interest. In this implementation, 100 bootstrap samples were used for parameter estimation, with a 60% validation dataset, and exposure biomarkers were ranked using quartiles [56]. We assumed a negative exposure association for correct answers (CAs) and planning scores, and a positive exposure association for reaction time (RT), omissions (Om), errors (Err), naming speed in the Stroop test, Go/NoGo test, and BRIEF scores, to assess the adverse effects of chemical mixtures on neurodevelopmental indicators.

The final WQS index was incorporated into a regression model, adjusted for covariates, to evaluate the overall effect of the mixture on the outcomes of interest. For each significant association, the importance of estimated weights for exposure biomarkers was calculated, with a significance threshold set at 0.167 (1/6, based on the number of biomarkers). To ensure stability and approximate the repeated holdout strategy described by Tanner et al [57], the standard WQS analysis was repeated 100 times (rh = 100) to simulate a distribution of validated results (Table SE [31]).

Finally, considering that previous studies reported sex-specific effects of EDCs, cognitive scores and prenatal exposure levels of chemicals were analyzed separately for boys and girls.

All statistical analyses were performed using R software version 4.1.2 [58]. The miWQS package was used for WQS regression analysis [59], while the MIPCA package [60] was applied as a preliminary step for multiple imputation prior to PCA modeling with the FactoMineR package [61]. Statistical significance was set at a *P*-value of .05.

## Results

### Descriptive analyses

The general characteristics of the study sample are displayed in Table 1. In total, 55 participants were included in the analysis, 55% of whom were boys. All children were born at term without complication. The average age of the children at the time of testing was 5 years and 9 months. Mothers were, on average, 30 years old at delivery and generally did not smoke or consume alcohol during pregnancy. Half of the women were primiparous, and most parents included in the study had at least a degree in higher education.

**Table 1:** Demographic characteristics

Variable	N	N (%)	Mean	SD	Min	Q1	Med	Q3	Max
Sex (boy)	55	30 (55)							
Age during tests (years)	55		5.75	0.34	5.08	5.55	5.8	6.02	6.28
Maternal age at delivery (years)	55		30	5	20	28	30	32	42
Parity (multiparous)	55	27 (49)							
Alcohol during pregnancy (>1 serving/month)	55	3 (5)							
Breast feeding	55	46 (84)							
Smoking parents	55								
None		41 (75)							
Only the father		8 (15)							
Only the mother		9 (16)							
Parental educational level (>high school)	55								
Mother		39 (71)							
Father		34 (62)							

**Figure 2:**
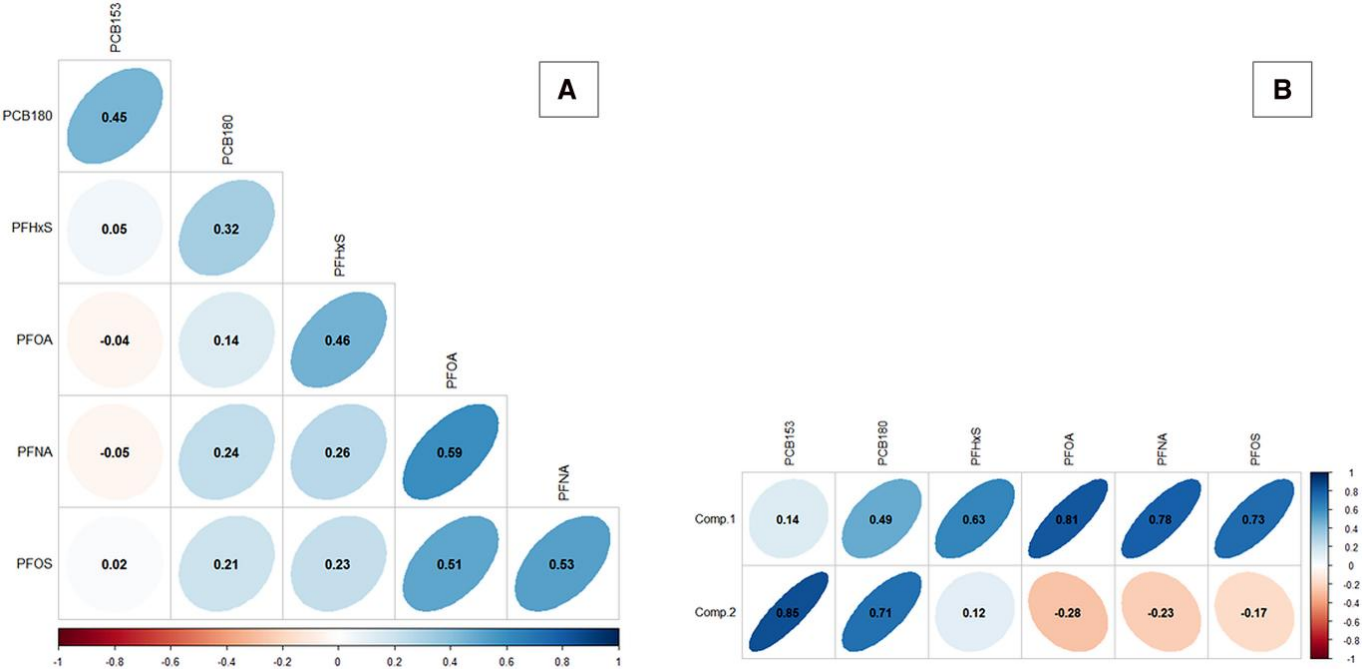
Pairwise Pearson's correlation coefficients between individual EDCs (A) and between the 2 components and toxicants (B); positive correlations are shifted to the right, whereas negative correlations are shifted to the left; the color coding is irrelevant.

Table 2 showed the distribution of EDCs in our sample, including the mean, median, and interquartile range of blood cord concentrations. Six chemicals were detectable in more than 50% of the study population. In our cohort, 84% of samples tested positive for biphenyl compounds including PCB-153 and PCB-180, which were present in 51% and 78% of the cases, respectively. Perfluoroalkyl substances had the highest detection rate (95%) with PFOA, PFNA, PFOS, and PFHxS detected in 89%, 80%, 76%, and 62% of the samples, respectively. As expected, some toxicants showed moderate positive correlation with each other (Fig. 2A). It should be noted that exposure levels to the different persistent organic pollutants (POPs) are independent of sex (Table SF [31]).

**Table 2:** Detection rate above the LOQ, geometric mean concentrations, SD, and quartiles of the EDCs considered in the study

Variable	N	N (%) > LOQ	LOQ (ng/mL)	Mean (ng/mL)	SD	Min (ng/mL)	Q1 (ng/mL)	Med (ng/mL)	Q3 (ng/mL)	Max (ng/mL)
PCBs										
PCB-153	55	28 (51)	0.07	0.05	0.05	<LOQ	<LOQ	0.07	0.08	0.19
PCB-180	55	43 (78)	0.05	0.05	0.03	<LOQ	0.05	0.06	0.07	0.18
PFASs										
PFHxS	55	34 (62)	0.15	0.19	0.21	<LOQ	<LOQ	0.17	0.27	0.94
PFOA	55	49 (89)	0.25	0.75	0.48	<LOQ	0.42	0.75	0.90	2.94
PFNA	55	44 (80)	0.10	0.18	0.13	<LOQ	0.11	0.17	0.24	0.53
PFOS	55	42 (76)	0.50	1.09	1.34	<LOQ	0.61	0.87	1.30	9.21

Concentrations were expressed in ng/mL.

Abbreviations: LOQ, limit of quantification; PCB, polychlorinated biphenyl; PFAS, perfluoroalkyl substance; PFHxS, perfluorohexane sulfonate; PFNA, perfluorononanoic acid; PFOA, perfluorooctanoic acid; PFOS, perfluorooctane sulfonate; SD, standard deviation.

The results of attentional and executive tests are summarized in Table 3. Correct answers, omissions (when the child failed to detect the target), errors (when the child responded when he did not have to, eg, in response to a distractor) were counted, and RTs (for correct responses) were measured for attentional, Stroop Fruits and Go/Nogo tests. The Spatial Planning test had a global score. Given the age of the children, there were no standards for attentional, Go/Nogo, and Spatial Planning tests. The scores, obtained in Stroop Fruits test fell within the age norms both for RTs and errors, across the different conditions [46] as were the scores of the BRIEF questionnaire (global score, indexes, and subscales). All children were able to name the color of the squares and the fruits.

**Table 3:** Mean and SD of neuropsychological tests results

Variable	N	Global score Mean (SD)	RT (ms) Mean (SD)	CA Mean (SD)	Err Mean (SD)	Om Mean (SD)
Attentional functions						
VSA	55		1280.30 (297.31)	10.62 (1.57)	2.51 (5.38)	1.36 (1.58)
ASA	55		2442.50 (415.00)	7.20 (2.93)	7.37 (7.36)	4.77 (2.89)
DA)	55		3452.92 (438.92)	12.08 (2.78)	5.55 (7.34)	3.95 (2.75)
Executive functions						
Naming speed (Stroop)	55		49.94 (12.09)		1.02 (1.27)	
Stroop inhibition	55		101.08 (31.06)		3.96 (3.47)	
Stroop flexibility	55		147.88 (55.35)		1.08 (1.10)	
Go/NoGo	55		799.01 (224.09)	25.91 (2.46)	4.84 (2.42)	3.93 (2.50)
Spatial Planning Test	55	14.20 (6.51)				
BRIEF	55					
Total score	55	45.98 (9.37)				
Behavioral Regulation Index	55	50.17 (9.07)				
Inhibition score	55	49.38 (9.84)				
Flexibility score	55	50.00 (8.71)				
Emotional control score	55	51.43 (9.96)				
Metacognition Index	55	43.89 (9.43)				
Initiation score	55	47.83 (11.08)				
Working memory score	55	48.89 (9.79)				
Planification score	55	40.57 (6.51)				
Organization score	55	53.26 (9.83)				
Auto-regulation score	55	44.19 (4.12)				

Abbreviations: ASA, Auditive Selective Attention; BRIEF, Behavior Rating Inventory of Executive Function; CA, correct answer; DA, Divided Attention; err, error; om, omission; RT, reaction time measured in microseconds; SD, standard deviation; VSA, Visual Selective Attention.

### EDCs and cognitive outcome

#### Principal components approach

The PCA allowed the exposome to be summarized into a few variables, known as components, which accounted for the correlations between the different toxicants. In our model, 2 main components were sufficient to describe 64% of the total variability, capturing 41% and 23% of the variance, respectively. The loading factors for each chemical on each component are presented in Fig. 2B. The first component (Comp 1) explained the largest portion of the variance and was characterized by high factor loadings for all PFASs and PCB-180. The second component (Comp 2) primarily reflected exposure to PCBs. In sex-stratified analyses, the distributions of components 1 and 2 appear to be relatively comparable.

Adjusted multivariate regression analysis conducted in the whole population revealed a positive association between Comp 2 and the omission rate in the DA test, reflecting lower performance (β [standard error, SE] = .47 [0.22], *P* = .031) (Table 4). No associations were found between any of the 2 components and the results of the VSA, ASA, Stroop, Go/NoGo, Spatial Planning tests or the global score of the BRIEF. Interaction models further identified sex-specific effects on EFs. In girls, Comp 1 was positively associated with the Behavioral Regulation Index of the BRIEF questionnaire (β [SE] = 3.71 [1.27], *P* = .012) indicating that higher exposure to Comp 1 is associated with lower level of behavioral control. This association was primarily driven by underlying association with Inhibition (β [SE] = 2.09 [0.45], *P* = .0002) and Emotional Control (β [SE] = 1.89 [0.67], *P* = .010) subscales. In boys, Comp 2 was positively associated with the Metacognition Index of the BRIEF questionnaire (β [SE] = 3.97 [1.70], *P* = .030), which was mainly driven by lower-level associations with Working Memory (β [SE] = .84 [0.32], *P* = .038) and Planning (β [SE] = .85 [0.39], *P* = .033) subscales.

**Table 4:** Adjusted multiple linear regressions between PCA components and WQS index and neuropsychological scores

	VSA			ASA			DA		
	RT β (SE)	Err β (SE)	Om β (SE)	RT β (SE)	Err β (SE)	Om β (SE)	RT β (SE)	Err β (SE)	Om β (SE)
Comp 1	9.14 (43.20)	1.36 (0.96)	.61 (0.38)	−3.88 (26.25)	−.11 (0.48)	.01 (0.15)	−70.16 (40.11)	−.06 (0.74)	−.20 (0.31)
Girls	39.55 (84.58)	.45 (1.01)	.44 (0.38)	−74.25 (39.07)	−.50 (0.88)	−.30 (0.28)	21.78 (35.48)	−.44 (1.47)	.16 (0.88)
Boys	−24.27 (40.94)	1.87 (0.98)	.70 (0.39)	51.44 (35.52)	.23 (0.56)	.25 (0.16)	−78.84 (45.97)	.33 (0.70)	−.31 (0.40)
Comp 2	−99.32 (59.44)	.66 (1.16)	−0.09 (0.83)	1.18 (35.20)	1.34 (0.71)	.09 (0.20)	65.38 (57.17)	1.59 (1.04)	**.47** (**0.22)**
Girls	−148.80 (114.10)	.81 (1.43)	−.07 (0.91)	9.21 (60.11)	2.07 (1.11)	.44 (0.36)	98.10 (71.20)	3.29 (1.87)	.02 (0.51)
Boys	−67.31 (53.80)	.55 (2.37)	−.37 (0.54)	−.17 (49.02)	.32 (0.89)	−.14 (0.20)	−11.96 (74.75)	.21 (0.98)	.55 (0.29)
WQS	115.76 (76.99)	3.49 (2.40)	1.49 (0.92)	−16.62 (51.20)	1.51 (0.98)	0.16 (0.33)	−126.45 (83.97)	2.51 (1.27)	**1.18** (**0.38)**
Girls	213.10 (119.60)	2.72 (1.72)	1.34 (0.87)	−132.82 (79.21)	2.89 (1.60)	.11 (0.53)	−122.50 (154.90)	4.65 (2.49)	1.13 (0.81)
Boys	−57.39 (91.42)	4.85 (2.49)	1.58 (0.89)	108.76 (61.03)	.56 (1.12)	.40 (0.29)	−133.10 (118.77)	2.18 (1.60)	**1.09** (**0.43)**

	Stroop inhibition	Stroop flexibility	Go/NoGo	Planification	BRIEF		
	Err β (SE)	Err β (SE)	Err β (SE)	Score β (SE)	Global score β (SE)	Behavior regulation β (SE)	Metacognition β (SE)
Comp 1	−.24 (0.32)	−.46 (0.45)	.12 (0.22)	.99 (0.57)	−.04 (2.05)	1.49 (0.82)	−.04 (1.35)
Girls	−.24 (0.45)	−.86 (0.80)	−.02 (0.40)	1.40 (0.89)	−4.50 (3.00)	**3.71 (1.27)**	−2.46 (2.06)
Boys	−.16 (0.43)	−.25 (0.56)	.12 (0.29)	.34 (0.73)	2.27 (3.26)	.44 (1.43)	**3.97 (1.70)**
Comp 2	.05 (0.42)	.63 (0.63)	−.01 (0.34)	−.42 (0.78)	3.82 (2.43)	−.20 (0.74)	3.55 (1.80)
Girls	−.09 (0.63)	.47 (1.10)	.47 (0.54)	−.15 (1.23)	6.04 (3.19)	−1.09 (1.34)	3.40 (2.42)
Boys	.05 (0.52)	.77 (0.80)	−.36 (0.44)	−.48 (1.05)	2.20 (4.49)	.48 (1.10)	4.29 (3.20)
WQS	−.10 (0.64)	−.08 (0.22)	.60 (0.47)	1.80 (1.25)	8.31 (4.46)	2.49 (1.53)	4.58 (2.53)
Girls	.03 (0.94)	−.50 (0.34)	.54 (0.90)	2.80 (2.09)	7.32 (4.92)	**4.52 (1.86)**	1.82 (3.57)
Boys	−.28 (0.82)	.16 (0.30)	.70 (0.60)	.01 (1.69)	9.07 (5.80)	1.87 (1.10)	**11.60 (3.54)**

Statistically significant results (*P* < .05) are in bold.

ASA, Auditive Selective Attention; BRIEF, Behavior Rating Inventory of Executive Function; CA, correct answer; DA, Divided Attention; Err, error; Om, omission; PCA, principal component analysis; RT, reaction time measured in microseconds; SE, standard error; VSA, Visual Selective Attention; WQS, Weighted Quantile Sum.

#### WQS model

Negative associations were also identified using the WQS approach (Table 4). For each significant correlation, the relative weight of each component of the mixture was evaluated and is presented in Table 5.

**Table 5:** Weights from Weighted Quantile Sum regression for pollutant index and risk of lower neuropsychological performances

Divided Attention				BRIEF indices			
More omissions Total population		More omissions Boys		Behavioral regulation Girls		Metacognition Boys	
Chemical	Weight	Chemical	Weight	Chemical	Weight	Chemical	Weight
**PCB-153**	**0**.**543**	**PCB-153**	**0**.**514**	**PCB-180**	**0**.**394**	**PCB-180**	**0**.**581**
**PCB-180**	**0**.**172**	**PCB-180**	**0**.**247**	**PFOS**	**0**.**219**	**PFOA**	**0**.**376**
PFHxS	0.110	PFHxS	0.093	**PFOA**	**0**.**195**	PCB-153	0.032
PFOA	0.086	PFOA	0.070	PFHxS	0.075	PFHxS	0.006
PFOS	0.051	PFNA	0.044	PFNA	0.064	PFOS	0.002
PFNA	0.038	PFOS	0.032	PCB-153	0.052	PFNA	0.002

Weights above 0.166 are in bold.

Abbreviations: BRIEF, Behavior Rating Inventory of Executive Function; PCB, polychlorinated biphenyl; PFHxS, perfluorohexane sulfonate; PFNA, perfluorononanoic acid; PFOA, perfluorooctanoic acid; PFOS, perfluorooctane sulfonate.

In the whole sample, a significant positive association was observed between prenatal exposure to EDCs and DA scores, indicating lower performance (β [SE] = 1.18 [0.38], *P* = .009). In this test, a greater number of omissions were observed in children exposed to a mixture predominantly loaded with PCBs.

In the sex-stratified analysis, the negative association with DA performance remained significant only in boys (β [SE] = 1.09 [0.43], *P* = .024). In girls, the positive correlation observed in the PCA model between antenatal EDCs exposure and the Behavioral Regulation Index of the BRIEF questionnaire was also confirmed in the WQS approach (β [SE] = 4.52 [1.86], *P* = .025). Finally, in boys, a positive correlation was observed between exposure to a mixture primarily loaded with PCB-180 and PFOA and the Metacognition Index of the BRIEF questionnaire (β [SE] = 11.60 [3.54], *P* = .005). Again, this result was mainly driven by underlying association with Working Memory (β [SE] = 3.44 [1.10], *P* = .007) and Planification (β [SE] = 3.82 (1.27), *P* = .008) subscales.

Consistent with the PCA results, no associations were found between the compounds and performance on the VSA, ASA, Stroop, Go/NoGo, and Spatial Planning tests, or with the total BRIEF score.

## Discussion

We assessed cord blood concentrations of 6 EDCs and investigated their combined effects on attentional and EFs in 6-year-old children. Despite relatively low exposure levels compared to other population-based studies [27, 29], our findings provide evidence of associations between antenatal exposure to a mixture of PCBs and PFASs and attentional and executive functioning in young children.

Both PCA and WQS models revealed that the number of omissions in the DA test is positively associated with the level of exposure to a mixture of EDCs. Polychlorinated biphenyl-153 and PCB-180 were the primary toxicants implicated in both approaches. In sex-stratified analyses, this association remained significant only in boys when assessed with the WQS approach, with similar toxicants identified.

Sex-specific analyses also showed significant associations between antenatal exposure to a mixture of EDCs and poorer EF skills, as measured by the BRIEF indexes, with effects differing by sex: antenatal exposure was negatively associated with the Behavioral Regulation Index in girls, while the Metacognition Index was adversely affected in boys.

### Impact of EDC mixture on attentional and EFs in children

Our analysis indicated a correlation between prenatal exposure to EDCs and DA across the whole sample using both PCA and WQS approaches. Exposure level to a PCB-dominant mixture is predictive of omission rates in the DA task. The increase in omissions on the DA test indicated poorer abilities to allocate attentional resources to different modalities, although there was no effect on RTs or on the number of errors. This result was in line with findings of Neugebauer et al [62], the only other team to study the impact of EDCs on DA using the KITAP, a computer-based test battery of attention performance.

To our knowledge, no prior study had analyzed the combined effects of PCBs and PFASs on attentional and EFs. Furthermore, the effects of antenatal exposure to PCBs or PFASs on attentional and EFs are inconsistent in the literature (Tables SA and SB [31]). To date, 41 studies from 22 different cohorts have examined associations of prenatal PCBs with behavioral regulation, child attention, and EF among children ranging mostly from ages 3 to 12 years. Among studies that analyzed clinical and ADHD-like symptoms, including ADHD diagnosis, the majority reported null associations with PCBs [63-73]. Six studies showed negative associations, including poorer impulse control [74], deficit in attention [74, 75], more hyperactivity [74, 76, 77], and externalizing problems [78, 79]. Conversely, 3 of them demonstrated less hyperactivity [62], externalizing [80], or problematic behaviors [81]. Compared with other study that also used specific neuropsychological tests to assess attentional performance, most reported a higher risk of attentional problems related to prenatal PCBs exposure. The attentional functions found to be negatively impacted are selective attention [82], DA [62], and sustained attention [83-86]. Only few studies, with various exposure levels, failed to find significant associations [63, 64, 72]. Furthermore, no effect on alertness has been found so far [62, 66, 87]. Executive functions were also examined with numerous specific tests demonstrating a deleterious impact of an antenatal exposure to PCBs on processing speed [82, 86, 88-91], working memory [82, 84, 89, 92-94], inhibition [82, 89, 92, 95-97], and planning [84]. Some studies also found no effect on prenatal exposure to PCBs on EFs [32, 62, 63, 66, 85]. Finally, only the studies by Berghuis et al [36] and Oppenheimer et al [33] demonstrated better performances in selective attention and working memory respectively, with tests carried out exclusively on teenagers.

Research on PFASs showed considerable heterogeneity with mixed findings. In studies using clinical and/or behavioral scales, the Strengths and Difficulties Questionnaire was frequently used to assess ADHD-like symptoms such as hyperactivity and attention difficulties. Some research linked antenatal exposure to PFASs with higher scores, indicating more behavioral problems [81, 98-102], while other studies found the opposite [71, 80, 102, 103]. Reflecting this inconsistency, Liew et al [104] reported both positive and negative associations between higher PFASs quartiles and ADHD-like symptoms in their cohort. Finally, although the HOME Study demonstrated more externalizing behaviors using the Behavioral Assessment System for Children questionnaire [105], several other studies reported either null [34, 68, 103, 106-111] or even negative associations [103, 109] to PFASs exposure.

Few studies specifically tested attentional skills. Among those, only Vuong et al [112] demonstrated better performance at the Conners’ Continuous Performance Test (CPT) in children more exposed to PFHxS. No additional association was found in other analyses by Vuong et al [105] or by Carrizosa et al [20] regarding sustained attention. Moreover, alertness, selective, and DA were not examined.

Lastly, studies examining the impact of antenatal PFASs exposure on EF demonstrated no effect on working memory [20, 35]. Results on metacognition are contradictory [102, 105], and those concerning inhibition are inconsistent since Vuong et al [112] demonstrated less inhibition while Carrizosa et al [20] showed no impact on this function.

Thus, our results were consistent with those of studies that reported a higher risk of attentional problems related to prenatal PCBs exposure [82-86]. Perfluoroalkyl substances had lower weights in the mixture and did not significantly impact attentional function, as reported in the recent review of Forns et al [29].

### Sex-stratified analysis

Several studies reported sex-specific links between cognitive outcomes and a number of environmental chemicals included in our analysis. However, no clear pattern emerged regarding the direction of associations for ADHD-like symptoms, attention, and EFs, with many results closed to null or oriented in both directions.

In the sex-stratified analyses of our study, the association with the DA test remained significant but only for boys, with both WQS and PCA models, showing more difficulties in distributing attentional resources across different modalities. Although no other studies to our knowledge analyzed the sex-specific effects on DA, these results were consistent with those of Sagiv et al [91] who found a higher rate of omissions in a CPT among boys exposed to PCBs. In the same study, girls had considerably shorter CPT RTs with increasing exposure, particularly for the sum of PCBs, highlighting a potential attentional vulnerability to PCBs in boys. However, these observations were not consistent with those of Stewart et al [95] and Berghuis et al [36] who demonstrated no sex difference in the impact of antenatal exposure to PCBs on sustain attention functioning.

In boys again, using both PCA and WQS approaches, positive correlations were found between a mixture of PCBs and PFASs, and the Metacognition Coefficient of the BRIEF questionnaire, reflecting a lower ability to manage cognitive processes effectively. More precisely, this result was mainly explained by lower scores on the Working Memory and Planification subtests. A sex-dependent impact on working memory was already demonstrated in our previous study on intellectual development: In the same cohort, significant positive association between Comp1 (ie, higher overall PFASs exposure) and better Working Memory Index scores of the Wechsler battery was observed only in girls, reflecting poorer performance among boys [24]. Several studies demonstrated the relationship between poor executive functioning and perinatal PFASs [20, 34, 102, 105, 112] or PCBs [82, 84, 89, 92, 94, 113] exposure. However, among these, only the HOME study [112] and the INMA Project [20] demonstrated a sex effect to the detriment of boys, while others did not [32-35, 91].

Finally, exclusively in girls, a positive correlation between antenatal EDCs exposure and the Behavioral Regulation Index of the BRIEF questionnaire was found in our study using both PCA and WQS approaches. This result reflected less ability to shift cognitive set and modulate emotions and behavior via appropriate inhibitory control. It was mainly explained by lower scores on the Inhibition and Emotional Control subtests that were all negatively influenced by exposure to a mixture of PCBs and PFASs.

Few studies examined the combined impact of EDCs on behavioral regulation, particularly inhibition, in girls. Although numerous studies demonstrated the adverse impact of antenatal exposure to PCBs on inhibition [82, 89, 92, 95-97], no sex differences were proven [32, 91, 92]. Nevertheless, the recent analysis of Forns et al [29], the largest collaborative effort to assess the association between PFASs and ADHD in 9 European population-based studies, suggested a potential differential effect of PFASs related to child sex with a possible increased prevalence of ADHD in girls associated with exposure to PFOS and PFOA. Other studies also suggested that girls exhibit a higher prevalence of ADHD-like symptoms than boys in relation to PFOA [109], PFNA [103], or mixture of PFASs [34] exposure. However, results were inconsistent as some studies reported higher risks for boys [20, 78, 102, 103, 105, 109], while others reported no association at all [20, 34, 35, 71, 102, 103, 109, 114], although the combined effect of PCBs and PFASs has not been analyzed elsewhere.

### Physiopathology

There is a lack of sufficient knowledge regarding the biological mechanisms underlying the observed effects of EDCs on attentional and EFs in humans [115]. Polychlorinated biphenyls can disrupt dopaminergic functions, as evidenced by alterations in dopamine levels in cell cultures and the brains of laboratory animals, particularly in the prefrontal regions, which are thought to be of particular importance in these higher-order functions [116, 117]. Other animal studies show that neonatal exposure to low doses of PFASs can also induce irreversible neurotoxic effects in adult mice and cause changes in behavior and habituation by altering the dopaminergic and cholinergic system [118, 119]. Furthermore, these chemicals can alter levels of neural proteins essential for the formation and growth of synapses [120]. These are possible mechanisms through which PCBs and PFASs may affect attention-related behaviors, as decreases in cellular dopamine have been correlated with attention disorders such as ADHD [121, 122].

Moreover, EDCs increase the risk of childhood neurodevelopmental disorders by interfering with hormone signaling or metabolism. The thyroid axis is a major target due to its involvement in neuronal migration, synaptogenesis, and myelination during gestation and early childhood [123].

Finally, our results, along with those of previous studies, report sex differences in antenatal exposure to EDCs and child health, emphasizing the idea that early-life exposure to environmental toxicants has different health effects on boys’ and girls’ cognitive development. The mechanisms of action underlying these sex-specific associations are not fully understood, but prenatal exposure PFASs [124] and PCBs [125] has been linked to effects on the steroid axis, which plays a critical role in brain organization of the neuroendocrine circuitry that coordinates sex-specific physiology. Numerous neurons express steroid hormone receptors at different development stages, making them likely targets of chemicals [126]. Estrogen has been suggested as a critical protective factor, while perinatal exposure to androgens, particularly testosterone, modulates striatally based dopaminergic circuits in such a way as to place boys at greater risk for early developing inattention disorders [29]. Thus, the endocrine-disrupting effect of PFASs and PCBs on steroid signaling in the brain could impact cognition in a sex-dependent manner [127]. In addition, many other biological mechanisms may explain the heightened vulnerabilities of the male brain to toxics, compared to the female brain. Some animal and human studies have shown differences especially in oxidative processes, neuroprotection phenomena, and EDCs pharmacokinetics (accumulation, distribution, and clearance) between males and females [127, 128]. Finally, some epigenetic mechanisms are hormonally regulated and may modulate the effects of early life EDCs exposures on long-term health outcomes [128].

### Strength and limitations

Our study has several strengths, including its prospective design. Antenatal EDCs exposure was evaluated using cord blood, which is an excellent matrix for monitoring chemical exposures in newborns. The long half-life of these chemicals in humans indicates a steady exposure condition during pregnancy. As such, a single cord blood measure of these compounds has been suggested as a good indicator of fetal exposure [129]. Moreover, studies have shown that gestation and early life may be the most sensitive period of neurodevelopment [26], when the central nervous system is rapidly developing. We used standardized tests with trained examiners to assess key attentional and executive outcomes. Furthermore, attentional and EFs were mainly evaluated using objective measures, rather than relying on parent or teacher questionnaires, which provide indirect subjective measures of cognitive outcomes. Finally, 2 validated and complementary statistical approaches were employed to study the joint effect of a mixture of EDCs, considering the main confounding factors. The selection of relevant chemicals and the use of PCA and WQS reduced the number of variables studied and, therefore, the risk of chance association.

The main limitations of this study include the relatively small sample size and low participation rate in the context of Covid pandemic, which may introduce selection bias. The results of the present study need to be confirmed with a larger cohort. However, a comparison between the initially enrolled population (N = 212) and the subset tested shows that the characteristics are generally comparable (Table SG [31]). Measurements of the different POPs, particularly PCBs, in our study are reported on a wet-weight basis. The absence of lipid adjustment may increase measurement error in estimating the individual's true body burden. Indeed, PCBs are highly lipophilic compounds that bind strongly to circulating lipids, which vary according to several factors, including age, sex, body mass index, and fasting status at the time of sampling. Without lipid correction, comparisons of wet-weight PCB concentrations between groups may therefore be misleading [130]. However, the lipid profile of cord blood (particularly its composition, variability, and metabolism) differs from that of adults, and the relationship between PCBs and lipids in this biological matrix has not been clearly characterized [131, 132]. In addition, cord blood sampling depends on delivery conditions. When samples are not collected under standardized fasting protocols, or when lipid concentrations exhibit substantial variability unrelated to exposure, lipid adjustment may introduce bias through either dilution or amplification effects [133]. Consequently, lipid concentrations may themselves be altered by PCB exposure [134] or associated with other risk factors [135]. In such cases, adjusting for lipids or normalizing PCB concentrations by lipid levels may introduce bias, including collider bias. Finally, numerous cohort studies, as well as several meta-analyses, have opted for and justified the use of unadjusted (wet-weight) concentrations [86, 92, 136]. Our results may also have been influenced by the contributions of others toxicants not considered, such as methylmercury [86] or lead [62]. Conversely, it has been suggested that some seafood-related contaminants, such as PFASs, could serve as a proxies for fish nutrients, showing protective associations that may actually relate to the benefits of seafood consumption [20]. Furthermore, attentional testing is not validated for children under 6 years of age. Indeed, the diagnosis of ADHD is uncertain in preschool children, as only 50% of those diagnosed before age 4 will still meet the diagnostic criteria at age 6 [137]. However, our analysis does not address the diagnosis of ADHD but rather the efficiency of attentional functions involving various level of selectivity, in accordance with the Van Zomeren and Brouwer model [1]. The attentional components varying along the dimension of intensity, such as alertness and sustained attention have not been examined in our study, although it is notably affected by antenatal exposure to certain toxicants, especially PCBs, as reported in the literature. The use of different neuropsychological tests has limited the comparability of existing birth cohort studies, in addition to the variation exposure doses and timing. Finally, it is worth noting that both attentional and EFs influence a broad range of domains, including academic achievement and social relationships [5, 6], which may constitute valuable outcomes for future research.

Differences in the relative proportion of targets vs distractors across attentional tests may partially explain discrepancies in study findings, as this ratio influences attentional effort and response bias, which, in turn, may affect the likelihood of making omission errors or other mistakes [138]. Discrepancies may arise between results obtained from neuropsychological tests and questionnaire-based assessments within the same study population. Unlike neuropsychological tests, which are designed to provide an more objective assessment of underlying cognitive functions, questionnaires assess the efficiency of EFs in everyday life as far as can be evaluated by an untrained external observer. Moreover, given that EFs are highly interrelated, some authors argue that they cannot be easily dissociated [139]. Differences between how confounding variables were controlled may also explain some inconsistencies in other studies. For example, breastfeeding is not accounted for in all studies, even though it has an important protective effect on attentional capacities [82, 140]. On the other hand, children exposed to some EDCs postnatally via breastfeeding may show more severe deficits later in life compared with no-breastfed children [69]. Another example is the consideration of socio-economic status, which we assessed here by parental education level. Indeed, there is evidence linking EDCs exposure to poorer neuropsychological scores, particularly among those with greater prenatal social disadvantage [23, 33].

Finally, our approach to sex-stratified analyses was descriptive and exploratory in nature, aiming to identify potential differential trends between boys and girls. Given the overlap of confidence intervals, any conclusions regarding sex-specific effects should be interpreted with caution. However, the limited sample size and the low number of events in certain subgroups would likely have resulted in unstable and unreliable estimates had we formally tested for effect measure modification using interaction terms.

## Conclusion

In the present study, early-life exposure to mixtures was significantly associated with the attentional and EFs of preschool children.

Our findings also support the hypothesis of a sex-specific effect, emphasizing that mechanisms involved may be sex-dependent. However, results should be interpreted with caution due to the small sample size.

## Data Availability

All datasets analyzed during the current study are not publicly available but are available from the corresponding author on reasonable request.
